# Rheostatic Regulation of the SERCA/Phospholamban Membrane Protein Complex Using Non-Coding RNA and Single-Stranded DNA oligonucleotides

**DOI:** 10.1038/srep13000

**Published:** 2015-08-21

**Authors:** Kailey J. Soller, Raffaello Verardi, Meng Jing, Neha Abrol, Jing Yang, Naomi Walsh, Vitaly V. Vostrikov, Seth L. Robia, Michael T. Bowser, Gianluigi Veglia

**Affiliations:** 1 Department of Chemistry, University of Minnesota, Minneapolis, Minnesota 55455.; 2 Department of Biochemistry, Molecular Biology, and Biophysics, University of Minnesota, Minneapolis, Minnesota 55455; 3 Department of Cell and Molecular Physiology, Stritch School of Medicine, Loyola University Chicago, 60153.

## Abstract

The membrane protein complex between sarco(endo)plasmic reticulum Ca^2+^-ATPase (SERCA) and phospholamban (PLN) is a prime therapeutic target for reversing cardiac contractile dysfunctions caused by calcium mishandling. So far, however, efforts to develop drugs specific for this protein complex have failed. Here, we show that non-coding RNAs and single-stranded DNAs (ssDNAs) interact with and regulate the function of the SERCA/PLN complex in a tunable manner. Both in HEK cells expressing the SERCA/PLN complex, as well as in cardiac sarcoplasmic reticulum preparations, these short oligonucleotides bind and reverse PLN’s inhibitory effects on SERCA, increasing the ATPase’s apparent Ca^2+^ affinity. Solid-state NMR experiments revealed that ssDNA interacts with PLN specifically, shifting the conformational equilibrium of the SERCA/PLN complex from an inhibitory to a non-inhibitory state. Importantly, we achieved rheostatic control of SERCA function by modulating the length of ssDNAs. Since restoration of Ca^2+^ flux to physiological levels represents a viable therapeutic avenue for cardiomyopathies, our results suggest that oligonucleotide-based drugs could be used to fine-tune SERCA function to counterbalance the extent of the pathological insults.

Heart failure is the leading cause of death worldwide^1^. Despite important advances in pharmacological and device therapies, the incidence and the financial impact of this devastating condition is increasing^1^. Current drug therapies aim to manage and ameliorate patients’ symptoms rather than cure the disease, which has generated a growing emphasis on the development of alternative approaches, including gene therapy^2^,^3^. As heart failure is a multifactorial event, several different strategies can be undertaken to reverse the decline of cardiac function^3^. One possible approach is to improve cardiac contractility by targeting calcium-handling proteins^2^,^4^. An emerging target for gene therapy is the membrane protein complex between the sarco(endo)plasmic reticulum Ca^2+^ -ATPase (SERCA) and phospholamban (PLN). The SERCA/PLN complex is a central regulator of cardiac contractility, transporting approximately 70% of the Ca^2+^ ions in the human heart^5^,^6^. SERCA reuptakes Ca^2+^ ions from the cytosol into the sarcoplasmic reticulum lumen during diastole^5^. PLN, a 52 amino acid transmembrane protein, controls SERCA’s apparent Ca^2+^ affinity, reducing its ability to transport Ca^2+^ when unphosphorylated and augmenting it when phosphorylated at Ser16 by protein kinase A^6^. Aberrant interactions between SERCA and PLN mutants and concomitant Ca^2+^ mishandling have been correlated with dysfunctional contractility and heart disease^7^,^8^. As a result, several approaches have been pursued to reverse these conditions; not only SERCA gene transfer therapy^4^,^9^,^10^, but also siRNA^11^, miRNA inhibition^12^ and aptamers^13^,^14^, have shown promise as therapeutic avenues. In particular, SERCA-directed gene therapy is the most effective strategy to augment Ca^2+^ transport and muscle contractility, either using an adeno-associated virus to overexpress the ATPase in cardiomyocytes or targeting PLN to reverse SERCA inhibition^10^. While the former approach is being evaluated in clinical trials, the latter presents challenges. In fact, PLN-knockout mice^15^ and PLN-null mutations in humans^16^ progress to heart failure and lethal dilated cardiomyopathy, respectively. Although encouraging for large animal models^17^, gene transfer of PLN^S16E^, a pseudo-phosphorylated mutant, hampers β-adrenergic control of heart contractility as the mutant can no longer be phosphorylated. In addition, current gene therapy methods do not allow a controllable response to counteract the different degrees of heart disease manifestations^4^. Whereas phosphorylatable mutants mimicking the phosphorylated state of PLN are being developed^18^,^19^, there is a critical need to devise more direct, viable methods to target PLN.

Here, we report the unexpected discovery that small RNAs and single-stranded DNAs (ssDNAs) bind with low nanomolar dissociation constants (*K*_d_) to the SERCA/PLN complex, regulating the ATPase’s apparent Ca^2+^ affinity in a tunable manner. Specifically, we found that both short RNAs and ssDNAs are able to reverse PLN’s inhibitory effects irrespective of their primary sequence. The functional effects are tunable by increasing or decreasing the oligonucleotides’ lengths, becoming significant for sizes encompassing typical endogenous miRNAs and reaching a plateau at 80 bases. Solid-state NMR and fluorescence spectroscopy data show that ssDNA binds PLN’s cytoplasmic domain specifically, but does not affect SERCA in the absence of the regulatory protein. In particular, NMR spectra show that ssDNA shifts the conformational equilibrium of the SERCA/PLN complex from inhibitory to a non-inhibitory state. Förster resonance energy transfer (FRET) experiments in HEK cells overexpressing the SERCA/PLN complex show that PLN remains bound to SERCA upon interacting with ssDNA. These functional effects, tested in membrane reconstituted systems, are reproducible in mammalian sarcoplasmic reticulum (SR) cardiac preparations. These findings indicate that chemically modified, non-coding RNA and single-stranded DNA templates with low off-target propensity can be exploited for developing compounds to target SERCA inhibition by PLN, thereby regulating Ca^2+^ transport in the SR. The rheostatic control of SERCA function achieved here opens up new possibilities for devising a graded response to varying extents of cardiac pathologies^10^.

## Results

### Small RNAs and ssDNAs bind PLN with low nanomolar dissociation constants, reversing its inhibitory effect on SERCA

**Figure 1:**
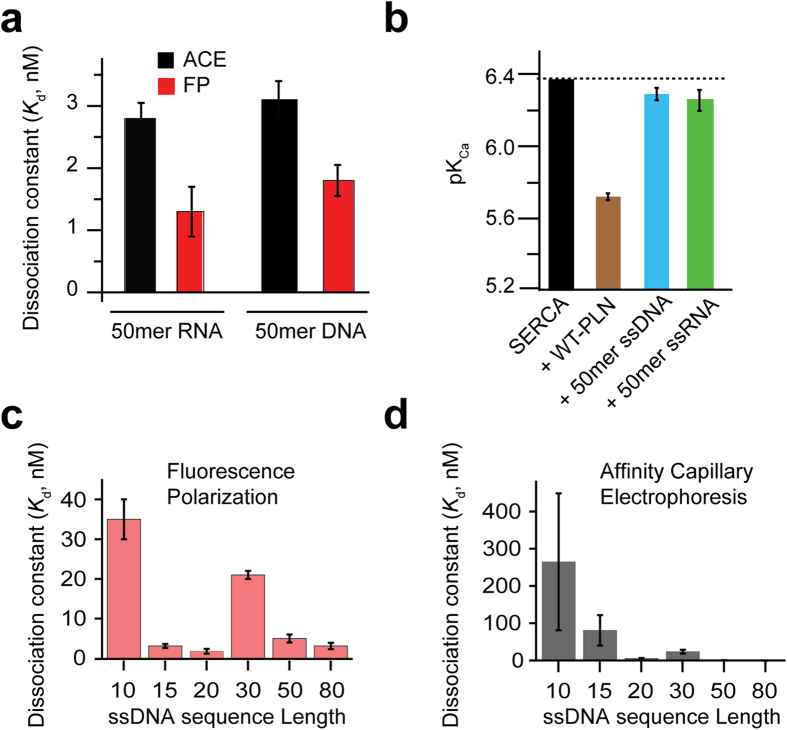
Functional effect of RNA and ssDNA on the SERCA/PLN complex. (**a**) Dissociation constants as measured by both fluorescence polarization and affinity capillary electrophoresis of both RNA and ssDNA. (**b**) pK_Ca_ of SERCA as measured by coupled enzyme assays. Dissociation constants versus ssDNA length obtained from fluorescence polarization (**c**) and affinity capillary electrophoresis (**d**). Sequences for (c) and (d) are found in Table 1.

Using affinity capillary electrophoresis (ACE), fluorescence polarization (FP), and native gel mobility shift assays, we found that short RNAs, of similar length to naturally occurring miRNA sequences, display low nanomolar dissociation constants for PLN. As an example, we report the dissociation constant of a random sequence RNA (50mer) to PLN in Fig. 1A and typical binding curves (80mer) in Fig. S1. Importantly, we found that these short RNAs not only bind, but also reverse PLN’s inhibition of SERCA. Fig. 1B shows the effect of the 50mer RNA on SERCA activity as monitored using coupled enzyme assays. The Ca^2+^ concentration at half maximal activity in the coupled assay curves indicates SERCA’s apparent affinity for Ca^2+^ ions (pK_Ca_). When SERCA is bound to PLN^WT^, the ATPase activity decreases and the normalized curves show a concomitant reduction of the pK_Ca_ value. Upon binding the 50mer RNA, SERCA’s function is nearly restored (Fig. 1B), mimicking the effect of PLN phosphorylation at Ser16^20^. Since aptamers have been selected to target specific proteins without off-target effects on gene expression^21^, we tested the effects of a 50mer ssDNA random sequence. Indeed, we found that both short RNAs and ssDNAs have similar *K*_d_ values for PLN (Fig. 1A) and restoration of SERCA function (Fig. 1B).

We also assessed the affinity of different lengths of ssDNAs with sequences chosen at random that had no significant secondary structures, with the exception of the 30mer (Table 1 and Fig. 1C,D). We found sequence length to be the best indicator of affinity. Any sequence containing more than 10 bases had low nanomolar *K*_d_ values, while sequences containing 10 bases or less had significantly less affinity with higher *K*_d_ values. Surprisingly, all of the randomly chosen sequences containing more than 10 bases exhibited high affinity for PLN, suggesting that this is a sequence-independent interaction (Table 1). The 80mer used for the binding studies was synthesized as a completely randomized mixture, with a 25% probability of each base being present at every register position. This random library allowed affinity to be determined independently of a specific sequence. The high affinity (low nanomolar *K*_d_) between the completely randomized sequence mixture of 80mer ssDNA and PLN confirms the sequence independence of this interaction. Among the longer sequences, the 30mer demonstrated slightly weaker affinity for PLN than predicted based on sequence length alone. This 30mer is the only sequence listed in Table 1 that exhibits significant secondary structure. The weaker than predicted affinity suggests a thermodynamic penalty to unfold the ssDNA before binding PLN. To analyze the nature of the intermolecular interactions, we carried out the binding assays at different salt concentrations (Fig. S2). Indeed, we found that the ssDNA/PLN interactions are persistent even at high salt concentrations and remain in the nanomolar range at NaCl concentrations up to 200 mM.

**Figure 2:**
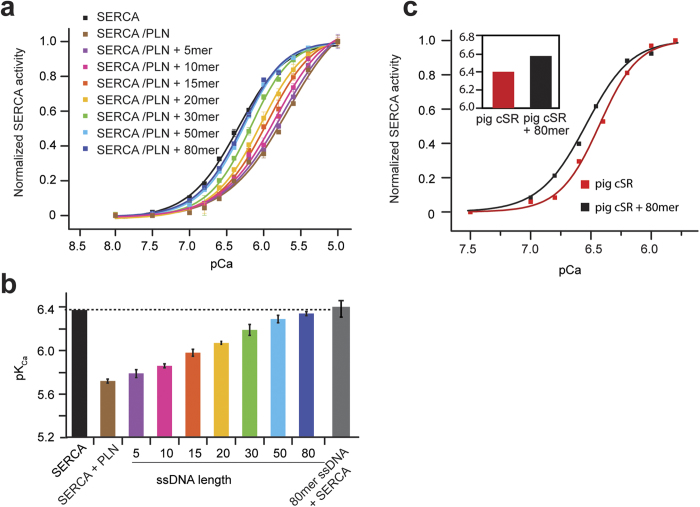
Tunable control of SERCA function using ssDNA of different lengths. Coupled enzyme assays in DOPC:DOPE lipid vesicles. (**a**) SERCA activity normalized as a function of Ca^2+^ concentration. Black is SERCA alone, brown is SERCA and PLN; other colors represent addition of different lengths of ssDNA. (**b**) pK_Ca_ values for the different ssDNA lengths derived from the pCa values at half maximum activity of SERCA. (**c**) Effects of ssDNA on pig cardiac SR preparations.

We then performed ATPase assays with varying length oligonucleotides and found they give rise to a graded effect on ATPase activity. Figure 2A depicts the normalized SERCA/PLN activity curves upon addition of ssDNA at different lengths and varying concentrations of free calcium (pCa). In the absence of ssDNA, PLN binding results in decreased Ca^2+^ affinity of SERCA (lowest pK_Ca_, with the brown curve shifted furthest to the right in Fig. 2A). Addition of ssDNA to the PLN/SERCA complex shifts the activity curve toward the higher pCa, indicating that the SERCA’s apparent affinity for Ca^2+^ ions is increasing; ssDNA reverses the inhibitory effect of PLN (Fig. 2A,B). The functional effect trends with sequence length, but not necessarily according to the relative affinity of the oligonucleotides (*K*_d_ values). This is evidenced by the 30mer, which displays a higher *K*_d_ than expected based on length alone, but still follows the length trend seen in the activity assays (Fig. 2A,B). It should be noted that all ATPase assays were performed at saturating ssDNA concentrations. Thus, the length trend (Fig. 2) observed is structural in origin and independent of the ssDNA concentration. Remarkably, longer oligonucleotides result in complete inhibition relief, an effect that mimics phosphorylation of PLN at Ser16 by protein kinase A^22^. With oligonucleotide sequences longer than 50 bases, we observed no further increase of SERCA activity beyond the physiological window, indicating that the effect is mediated by the direct interaction between ssDNA and PLN.

To confirm the specificity of the ssDNA/PLN interactions, we incubated 80mer ssDNA with SERCA in the absence of PLN. Under these conditions, the apparent Ca^2+^ affinity was identical to that of SERCA alone, confirming that ssDNA does not have an activating effect without PLN (Fig. 2B). Additionally, both double-stranded DNA and a mixture of free deoxynucleotides (dNTP) show no effect on PLN’s inhibition of SERCA; the pK_Ca_ with either double-stranded DNA or free dNTPs is identical to the pK_Ca_ of the PLN inhibited pump (Fig. S3). Taken together, these data suggest that reversal of the inhibitory effect of ssDNAs is manifested in a length-dependent manner, with inhibition relief being progressively more effective with increasing sequence length and reaching a plateau at ~80 oligonucleotides.

### ssDNA disrupts PLN inhibition without dissociating the SERCA/PLN complex

**Figure 3:**
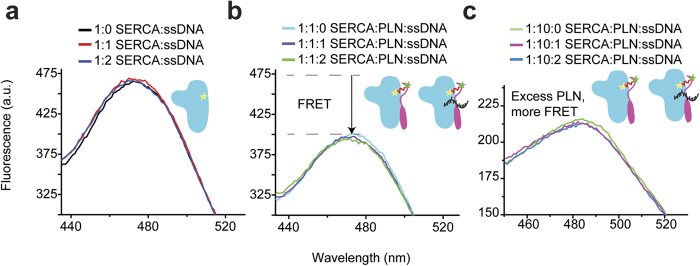
ssDNA does not dissociate PLN from SERCA. (**a**) Fluorescence spectra of SERCA alone and upon addition of ssDNA. (**b**) FRET of SERCA-PLN complex (cyan) in the absence and presence of ssDNA. Upon addition of ssDNA (purple and green) the FRET signal does not change, indicating no structural changes within the range of the FRET probes. (**c**) SERCA-PLN FRET at a 10:1 PLN:SERCA ratio (green).

To probe the specific interactions between PLN and ssDNA both in the absence and presence of SERCA, we labeled SERCA with a FRET donor (AEDANS) at position Cys674 and PLN with a non-fluorescent acceptor (Dabcyl-SE) on Lys3. FRET sample preparation matched that of the coupled enzyme assay samples (see Methods). In the presence of the FRET pair, we detected a marked decrease in fluorescence signal upon formation of the complex between SERCA and PLN (Fig. 3B). Addition of an equimolar amount of 40mer ssDNA (Table 1) to the pre-formed SERCA/PLN complex under conditions similar to the activity assays did not change the FRET signal. These measurements were repeated with excess PLN, or excess ssDNA, and the results were similar to the FRET data with stoichiometrical amounts (Fig. 3B,C). The enhanced FRET and shift of the emission maximum seen with a 10-fold excess of PLN is indicative of more FRET between SERCA and PLN, resulting from saturating the PLN binding site in the ATPase (Fig. 3C). We have also recorded fluorescence of SERCA^AEDANS^ without PLN, but in the presence of ssDNA. The fluorescence intensity was identical to that of SERCA^AEDANS^, illustrating that ssDNA does not interact with the ATPase alone (Fig. 3A). The FRET, ACE and fluorescence polarization data collectively demonstrate that the ssDNA interacts specifically with PLN irrespective of the presence of SERCA. Furthermore, ssDNA relieves PLN inhibition by causing a structural rearrangement of PLN without dissociating it from SERCA, in a manner resembling PLN phosphorylation at Ser16 – the endogenous mechanism for inhibition relief.

### ssDNA interacts with the SERCA/PLN complex in living cells

**Figure 4:**
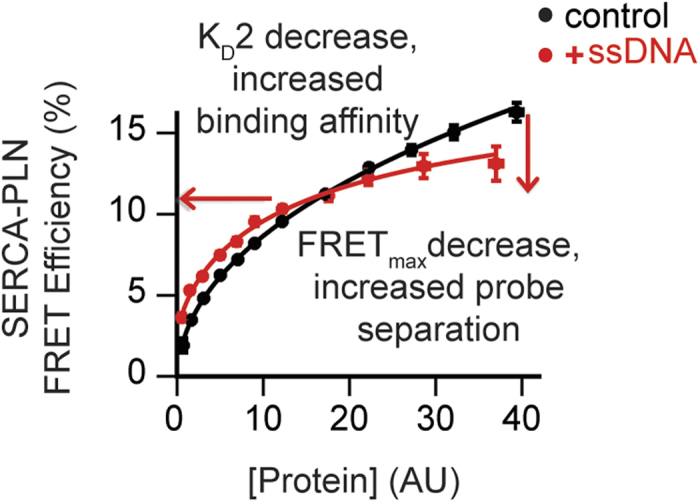
FRET experiments in living cells. ssDNA increased the affinity of the SERCA/PLN interaction, and decreased FRET_max_. ssDNA induces a conformational change of the complex, increasing fluorescent probe separation distance.

The SERCA/PLN interaction studies were also carried out in HEK cells. Specifically, FRET in-cell assays were used to investigate the interactions of ssDNA with free PLN and in complex with SERCA^23^,^24^. We utilized PLN^AFA^, a pentamer-destabilizing mutant with full inhibitory activity to minimize PLN oligomerization and detect small changes in binding affinity. Although it runs as a monomer on SDS-PAGE, PLN^AFA^ has a slight tendency to form pentamers in membranes and exhibits a significant intra-pentameric FRET when overexpressed in living cells. A cell-by-cell comparison of FRET with PLN expression level revealed a hyperbolic dependence of FRET efficiency on protein concentration for a population of HEK cells expressing the fluorescent proteins Cer-PLN^AFA^ and YFP-PLN^AFA^, fused at the N-terminus of PLN (Fig. S4). From this curve, we calculated the apparent dissociation constant for oligomerization (K_D_1) as well as the intrinsic FRET efficiency of the PLN oligomer (FRET_max_), reporting on the inter-protomer binding affinity and quaternary structure changes, respectively. In the absence of ssDNA, we found that PLN^AFA^ is able to form pentamers, though with reduced propensity compared to PLN^WT^. In contrast, addition of the 50-mer ssDNA to the cells containing plasmids encoding for Cer-PLN^AFA^ and YFP-PLN^AFA^ induced a notable increase in the apparent PLN-PLN affinity, representing an approximately 20% increase in PLN oligomerization (Fig. S4). A significant decrease in FRET_max_ was observed, which corresponds to an increase of the donor-acceptor distance (i.e., increased interprotomer affinity) as previously observed for PLN^R9C^ and its phosphomimetic mutants^25^,^26^. For the SERCA/PLN complex, cell transfection with 50-mer ssDNA had striking effects both on the binding affinity and the structure of the complex. Unlike the *in vitro* fluorescence experiments in which the AEDANS donor probe was attached at Cys674 in the SERCA’s P-domain, the Cer probe placed on the A-domain of SERCA is able to detect a four-fold increase of PLN affinity for SERCA upon oligonucleotide addition, with a concomitant decrease of SERCA/PLN FRET_max_ (Fig. 4). Taken with the *in vitro* ATPase activity assays, these data demonstrate that ssDNA binding to PLN mimics both the structural and functional effects of phosphorylation on the SERCA/PLN regulatory complex. The decrease of FRET_max_ upon complex formation is suggestive of a structural rearrangement of PLN within the complex; rather than the dissociation of PLN from the ATPase.

### ssDNA reverses PLN’s inhibition of SERCA in cardiac SR preparations

To assess the reproducibility of the ssDNA effects observed in the reconstituted systems in more biologically relevant conditions, we performed the activity assays using crude cardiac SR preparations isolated from pig ventricles, containing the SERCA2a isoform of the ATPase (see Material and Methods). Since PLN is endogenously expressed in ventricles, direct addition of ssDNA to the heavy SR preparations containing the SERCA2a/PLN complex would be expected to increase the ATPase apparent Ca^2+^ affinity. Indeed, addition of ssDNA (1 μM 80mer) to the pig crude SR vesicles augmented the pK_Ca_ by 0.15 (Fig. 2C). While the effect of ssDNA on SERCA activity in pig crude SR is somewhat lower than in the reconstituted system; this slight discrepancy is likely due to the challenges in determining the exact amounts of the SERCA/PLN complex in these native preparations, as well as their molar ratio and phosphorylation state. Overall, these data confirm the efficacy of ssDNA in relieving PLN inhibition of the mammalian SERCA2a/PLN complex under native conditions.

### NMR mapping of ssDNA binding epitope on PLN

**Figure 5:**
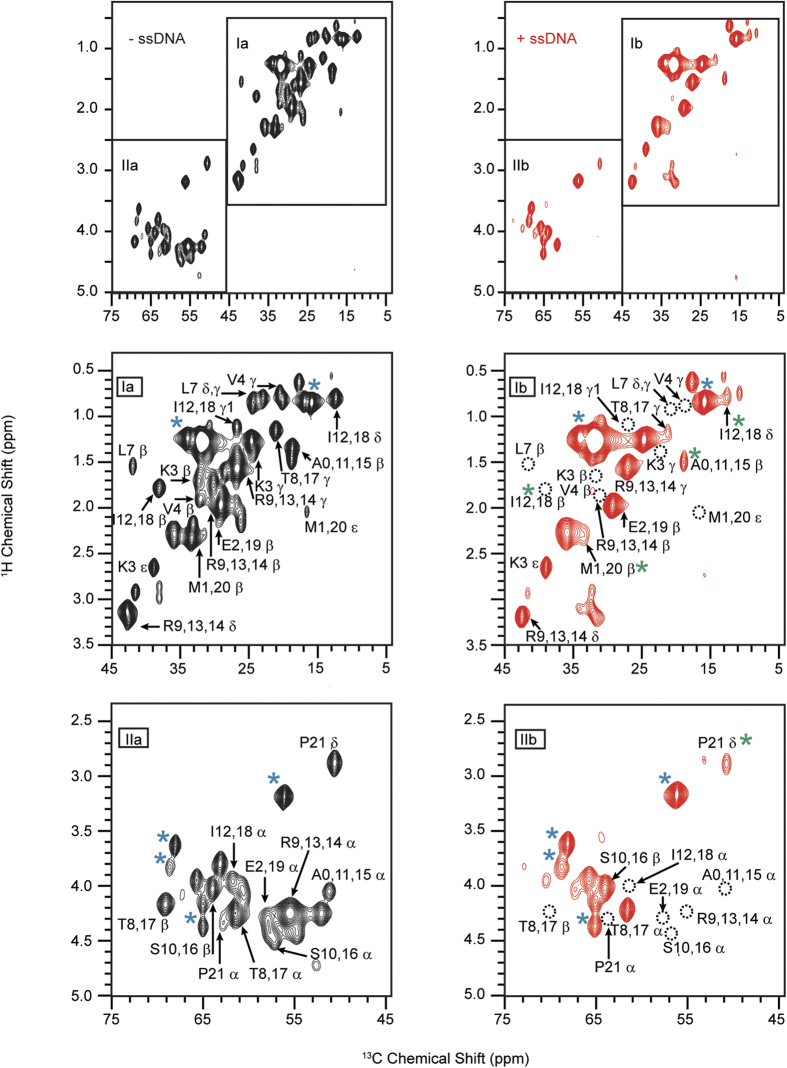
Mapping of PLN residues involved in ssDNA binding. [^1^H, ^13^C] RINEPT spectra of PLN in DMPC lipid vesicles in the absence (black) and presence (red) of ssDNA (80mer). Blue asterisks indicate lipid signals; green asterisks indicate a change in peak shape. Dotted circles indicate missing peaks.

To identify the specific residues of PLN interacting with the ssDNA, we used solid-state NMR (ssNMR) spectroscopy. We reconstituted U-^13^C/^15^N labeled PLN in deuterated DMPC lipid vesicles and monitored the chemical shifts of the backbone and side chain ^13^C resonances in the presence and absence of ssDNA (80mer). To detect the dynamic cytoplasmic domain of PLN, we used the refocused [^1^H, ^13^C]-RINEPT experiment^27^, which is well suited for protein domains undergoing fast reorientation (cytoplasmic domain of PLN) and insensitive to rigid domains on the NMR time scale (PLN’s transmembrane domain)^28^. In the free form of PLN, the resonances corresponding to the cytoplasmic region are all detectable. Addition of ssDNA to PLN at a 1:1 molar ratio causes the intensities of several amino acids peaks in the [^1^H, ^13^C]-RINEPT spectrum to decrease, with several peaks becoming broadened beyond detection (Fig. 5). The latter indicates the rigidification of PLN’s cytoplasmic domain and an increase in rotational correlation time upon ssDNA binding. In contrast, natural abundance ^13^C lipid signals are not affected by ssDNA and remain essentially unchanged (Fig. 5). The most affected resonances of PLN are located at the N-terminal portion of domain Ia (i.e., Lys3, Val4, Leu7, Thr8/17, Arg9/13/14, Ala/0/11/15, Ile12/18, and Glu2/19). To confirm that the ssDNA targets the cytoplasmic domain resonances, we compared the ssNMR data with solution NMR experiments carried out on PLN reconstituted in isotropic bicelles (q = 0.33). Due to the large size of the bicelle/PLN complex, solution NMR [^1^H, ^15^N]-HSQC experiments do not detect the transmembrane residues^29^, but provides visualization of the cytoplasmic domain resonances. Addition of 15mer ssDNA causes extensive line broadening (residues Glu2, Thr8, Ala11, Ile12/18 Arg13/14, and Met20 - Fig. S5), which is in qualitative agreement with ssNMR experiments. Taken with the FRET data, the NMR experiments indicate that the transmembrane domain of PLN remains essentially unperturbed and attached to the ATPase, while ssDNA primarily targets the cytoplasmic domain of PLN interfering with its regulatory function of SERCA.

**Figure 6:**
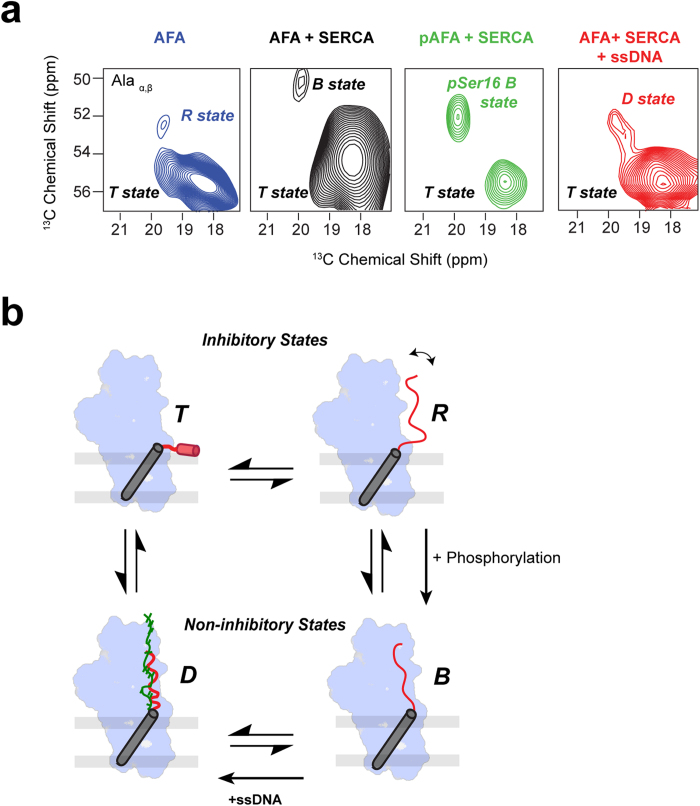
Effects of ssDNA on the conformational equilibrium of the SERCA/PLN complex (**a**) Portions of the [^13^C, ^13^C]-DARR spectra indicating the conformational equilibrium between the different states of PLN as probed by the alanine residues in the cytoplasmic domain. A small population of AFA PLN exists in the B (bound) state, upon phosphorylation, a higher population transitions to the B state (with a slight shift upon the phosphorylation), a further shift occurs upon addition of ssDNA to the D (DNA) bound state. (**b**) Proposed regulatory model of SERCA by PLN and effects of ssDNA. ssDNA shifts the monomer to pentamer equilibrium toward the oligomeric state. Phosphorylation of Ser16 shifts the equilibrium toward the non-inhibitory bound state (B-state). ssDNA is proposed to bind phospholamban and mimic the phosphorylated state, shifting the equilibrium toward a non-inhibitory D-state.

Previously, we found that PLN bound to SERCA undergoes conformational transitions between three major states (Fig. 6b): an inhibitory T state, with the transmembrane (TM) domain bound to SERCA and the cytoplasmic domain associated to the membrane; an inhibitory R state, with the TM domain bound to SERCA and the cytoplasmic domain unfolded and dissociated from the membrane; and a sparsely populated, non-inhibitory B or bound state, with both the TM and cytoplasmic domains interacting with SERCA^28^. PLN phosphorylation at Ser16 shifts the equilibrium toward the B state, undergoing a local structural rearrangement and effectively reversing PLN’s inhibition of SERCA. To assess the effect of ssDNA on the PLN conformational equilibrium in the presence of SERCA, we utilized PLN^AFA^, which simplifies the interpretation of the ssDNA binding on the SERCA/PLN complex by eliminating the monomer/pentamer equilibrium. To resolve the resonances in the aliphatic region of the spectrum, the cytosolic domain Ia of PLN^AFA^ was selectively labeled at 6 different sites (Val4, Leu7, Ala11, Ala15, Ile12, Ile18). These cytosolic residues (in the absence of SERCA) display chemical shift perturbations upon the addition of ssDNA, indicating binding between ssDNA and PLN^AFA^ (Fig. S6). Dipolar assisted rotational resonance (DARR) MAS [^13^C, ^13^C] experiments of U-^13^C/^15^N labeled PLN in complex with unlabeled SERCA were performed in the absence and presence of ssDNA (Fig. 6). Specifically, the DARR peaks corresponding to the Cα/Cβ correlations of the two Ala residues are sensitive to the conformational equilibrium between the different states and can be used as reporter residues (Fig. 6). In the absence of SERCA at 20 °C, these two residues populate a major conformational state (T state) and a sparsely populated R state. Upon addition of SERCA, there is a population shift toward the bound state that is augmented upon phosphorylation of PLN. Upon binding ssDNA, we observe a progressive shift of the two Ala residues of PLN^AFA^ toward lower fields, similar to what is observed with the non-inhibitory phosphorylated form of PLN. These results strongly support the fluorescence studies and show that ssDNA does not detach PLN from SERCA; rather it shifts the conformational equilibrium toward a non-inhibitory or D (DNA bound) state in a manner similar to the phosphorylated state. A hypothetical model of the conformational equilibrium of the SERCA/PLN complex and the effects of ssDNA is reported in Fig. 6B.

## Discussion

Homeostatic regulation of Ca^2+^ ions in muscle is crucial to proper contractility^5^. SERCA plays a central role in this delicate equilibrium and its function is regulated within a physiological window by PLN^28^. Super-inhibition of SERCA by PLN mutants or hyper-phosphorylation of PLN tip the homeostatic balance and lead to cardiomyopathies^2^,^30^. So far, there have been no drugs developed with a direct effect on the SERCA/PLN complex. However, the complexity of Ca^2+^ transport dysregulation in heart failure calls for therapeutic approaches that parallel the extent of the pathology, which is yet to be achieved.

Our study demonstrates that short RNAs and ssDNAs are able to regulate SERCA activity in a *tunable manner* by binding the cytoplasmic domain of PLN with nanomolar dissociation constants. Binding of ssDNA to the SERCA/PLN complex induces structural changes and promotes inhibition relief. Due to the high affinity of ssDNA for PLN, the tunability of the effects is not concentration-dependent, but rather length-dependent, with a range from 5 nucleotides, exhibiting minimal effects on SERCA activity, to 80 nucleotides, conferring a nearly complete relief of inhibition. The binding of the ssDNA is likely driven by the electrostatic interactions between the negatively charged phosphate groups of the oligonucleotide and the positively charged Lys and Arg side-chains of PLN. Such charge-charge interactions have been found in sequence-independent ssDNA binding proteins, such as T4 gp32, adeno-DBP and multiple others^31^,^32^,^33^. However, electrostatic interactions alone do not fully justify the unique binding seen between ssDNA and PLN. Single chain nucleic acids are essential for PLN binding, since neither double-stranded DNA nor a mixture of free dNTPs are able to reverse PLN inhibition of SERCA. ssDNA binds PLN without detaching it from the ATPase; rather, it shifts its conformational equilibrium toward a non-inhibitory state^18^,^28^. Previously, we found that PLN bound to SERCA undergoes conformational transitions between three major states: an inhibitory T state, with the transmembrane (TM) domain bound to SERCA and the cytoplasmic domain associated to the membrane; an inhibitory R state, with the TM domain bound to SERCA and the cytoplasmic domain unfolded and dissociated from the membrane; and a sparsely populated, non-inhibitory B or bound state, with both the TM and cytoplasmic domains interacting with SERCA^28^. By tracing the population of the SERCA-bound state of PLN, we found its conformational equilibrium is driven by ssDNA toward a new, distinct state (D state) (Fig. 6), which is non-inhibitory. Since FRET experiments show no detachments of PLN from SERCA, we surmise that the ssDNA may adopt a PLN-bound conformation with the phosphate backbone mimicking the effects of Ser16 and/or Thr17 phosphorylation and restoring SERCA’s apparent affinity for Ca^2+^ ions.

An important corollary to our study is the possibility of direct interactions of endogenous short RNA sequences (such as miRNA) with the SERCA/PLN complex. In the heart, miRNAs are involved both in cardiogenesis and disease including: hypertrophy, ischemia, and electrical remodeling^34^,^35^,^36^,^37^. miRNAs are up- or down-regulated in response to stress during cardiac disorders^37^ and they are notorious for their pleiotropic action, affecting multiple genes in different tissues^37^. The latter has prevented the progression of miRNA regulation to therapeutic applications^38^,^39^. Based on the high, non-sequence specific affinity of short RNAs for PLN, it is possible that endogenous miRNAs may have a role in cardiac pathophysiology that goes beyond gene regulation. As miRNAs of different lengths and sequences are produced for both protective and pathological roles^34^,^36^,^40^, the up-regulation of miRNAs under cardiac remodeling and heart failure may favor their direct physical interaction with the SERCA/PLN complex, affecting Ca^2+^ regulation and cardiac contractility.

Irrespective of the possible role of the endogenous miRNAs toward the SERCA/PLN complex in cardiomyocytes, the nanomolar affinity of ssDNA for PLN and its ability to reverse its inhibitory effects on SERCA constitutes a unique opportunity to exploit oligonucleotides as scaffolds for the design of small molecules to target Ca^2+^ regulation. While DNA aptamers or miRNA constructs have been successfully used to target and regulate the PLN gene^11^,^41^, silencing or ablation of the PLN gene has resulted in cardiomyopathies. In contrast, tunable regulation of PLN inhibitory function of SERCA by oligonucleotide-based drugs may represent a more promising therapeutic avenue in which the extent of SERCA activation can be fine-tuned to match the pathological insult.

## Methods

Wild type phospholamban expression and purification was achieved through the previously published methods^42^. Isotope labeling was accomplished via ^13^C-enriched glucose and ^15^N-enriched ammonium chloride. Calcium ATPase (SERCA1a isoform) was extracted from rabbit skeletal muscle according to previously reported procedure and purified by affinity chromatography using Reactive Red^43^. Pig cardiac SR vesicles (Lindenfelser’s Meats, Monticello, MN, USA) were prepared as reported previously^44^. Lipids were obtained from Avanti Polar Lipids (Alabaster, AL). Single stranded DNA (ssDNA) sequences were purchased from Integrated DNA Technologies, Inc. (Coralville, IA). Other chemicals were from Sigma Aldrich (St. Louis, MO).

### SERCA Activity Assays

Recombinant PLN was co-reconstituted with SERCA in multilamellar vesicles of 1,2-dioleyl-*sn*-glycero-3-phosphocholine (DOPC) and 1,2-dioleyl-*sn*-glycero-3-phosphoethanolamine (DOPE) at 4:1 DOPC:DOPE molar ratio. A solution of ssDNA in deionized water was added directly to the vesicles and incubated for 20 minutes at 25 °C prior to starting the assay. The molar ratio of the components were 700:10:1:1 (lipid : PLN : SERCA : DNA). Calcium dependence of the SERCA ATPase activity was measured at 25 °C using a coupled enzyme assay^45^ utilizing λ_340_ absorbance (NADH) as an analytical signal using a Spectromax microplate reader (Molecular Devices). The initial rate was measured as a function of calcium concentration, and the data were fit using the Hill equation (1):


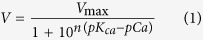


where: V is the initial rate; V_max_ is the maximum rate; pCa is the negative logarithm of calcium concentration; pK_Ca_ is the pCa value where V = V_max_/2, and n is Hill coefficient. For comparison, each data set was normalized to V_max_ after fitting. Inhibitory effects are expressed as changes in pK_Ca_ relative to SERCA alone.

Activity assays of SERCA2a were performed directly with pig cardiac SR, containing ~20 μg of total protein (bicinchoninic acid assay). Cardiac SR vesicles were diluted to 100 μl with buffer and incubated with 1 μM of ssDNA (80mer) for 30 min prior to measurements.

### NMR sample preparation

Magic angle spinning (MAS) NMR samples were prepared by co-dissolving 10 mg acyl chain deuterated 1,2-dimyristoyl-*sn*-glycero-3-phosphocholine (DMPC-*d*_*54*_) and 2 mg of recombinant [U-^13^C/^15^N]-PLN. The solvent was removed under a stream of nitrogen gas and desiccated overnight. The lipid/protein film was rehydrated with 1 ml of 20 mM MOPS, 100 mM NaCl (pH 7.0). The suspension was vortexed, briefly sonicated, lyophilized, re-suspended in 10 μL of ddH_2_O and transferred to a 3.2 mm thin wall MAS rotor. The final samples contained approximately 50% H_2_O (w/w) with a lipid:PLN ratio of 40:1. Magic angle spinning samples with PLN and SERCA were prepared by co-mixing the proteins at a 1:1 molar ratio in 1% C_12_E_8_, and adding them to 10 mg of DMPC-*d*_*54*_ solubilized with 1% C_12_E_8_. Next, ssDNA suspended in ddH2O was added and the detergent was removed by 3 hr incubation with BioBeads SM2 at a 30:1 Biobeads:C_12_E_8_ ratio at room temperature. The sample was diluted to 50 mL with buffer, pelleted by centrifugation at 4 °C (100,000 × *g*, 30 minutes) and the pellet was packed in the MAS rotor. Isotropic bicelle samples for solution NMR were prepared by dissolving 1 mg of [U-^13^C, ^15^N] PLN in 20 mM MOPS, 100 mM NaCl, and 5% D_2_O containing 174 mg/ml of 1,2-dihexanoyl-*sn*-glycero-3-phosphocholine (DHPC). The mixture was transferred to lyophilized DMPC (21.9 mg, corresponding to q = 0.33), vortexed, sonicated and subjected to several freeze/thaw cycles, until a clear solution was formed.

### NMR experiments

All NMR experiments were performed on a VNMRS spectrometer operating at ^1^H frequency of 600 MHz. Magic angle spinning (MAS) experiments were performed at 8 kHz spinning rate and at 25 °C, utilizing ^1^H/^13^C MAS probe (Varian). Pulse widths were 5.5 μsec (^13^C), 2.5 μsec (^1^H) and a 100 kHz ^1^H decoupling field strength was used. Two dimensional experiments were refocused Insensitive Nuclei Enhanced by Polarization Transfer (RINEPT) and Dipolar Assisted Rotational Resonance (DARR)^27^,^46^. A contact time of 1 ms was utilized for cross-polarization. Spectral widths were 100 kHz (direct dimension), 3.33 kHz (indirect dimension, RINEPT) and 40 kHz (indirect dimension, DARR). Chemical shifts were referenced to the ^13^CH_2_ signal of adamantane (40.48 ppm). Typically 640 scans with 30 increments in the indirect dimension were acquired. Data were processed using NMRPipe and analyzed with Sparky^47^.

### Affinity Capillary Electrophoresis (ACE)

The experiments were performed on a commercial CE system (P/ACE MDQ, Beckman Coulter, Inc., Fullerton, CA) with laser induced fluorescence (LIF) detection (λ_ex_ = 488 nm, λ_em_ = 520 nm). Binding and separation buffers for ACE and fluorescence polarization were made in nuclease free water (Coralville) and filtered through a 0.2 μm membrane filter (Millipore) before use. Separation buffer consisted of 20 mM MOPS and 0.1% (w/v) octaethylene glycol monododecyl ether (C_12_E_8_) at pH 7.0. Binding buffer additionally contained 0.25 mM DTT, 1 mM MgCl_2_, 1 mM KCl, and 5 mM CaCl_2_. Fluorescently labeled ssDNA samples in binding buffer (2.5 nM) were titrated with PLN and injected into a 50 cm × 50 μm fused silica capillary (Polymicro Technologies, Phoenix, AZ) using hydrodynamic injection (1 psi for 4 s). Separations were performed at reversed polarity, 30 kV for 10 min. Electropherograms were analyzed using Cutter 7.0; peak heights of the free ssDNA peaks were used to calculate the bound fractions according to the equation (2)^48^:


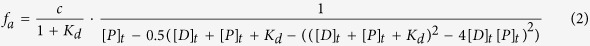


Where: f_a_ is the bound fraction; c is maximum bound fraction; [P]_t_ and [D]_t_ are total concentrations of PLN and DNA, respectively. Note that denominator represents the free concentration of PLN.

### Fluorescence Polarization (FP)

The experiments were performed on a Synergy^TM^ 2 Microplate Reader (BioTek Instruments, Inc., Winooski, VT) with similar filter settings (λ_ex_ = 485 ± 20 nm, λ_em_ = 528 ± 20 nm). Sample prepation was similar to the ACE experiments; 15 μL aliquots of mixtures were loaded into a corning 3540 microplate (Corning Incorporated, Corning, NY). Polarization values were calculated from the parallel and perpendicular fluorescence intensities. Calibration was done using the g factor, calculated in the Gen 5TM software (BioTek Instruments, Inc., Winooski, VT). Bound fractions were determined using equation (3):


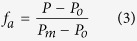


where: f_a_ is the bound fraction; P, P_m_ and P_o_ are measured polarizations of a sample, complex, and free ssDNA, respectively. The polarization of the complex (P_m_) was taken as the plateau with an excess of PLN. The overall fluorescence intensity of the samples with increasing PLN concentrations, was monitored and bound fraction was modified if the overall fluorescence intensity was positively or negatively biased according to the published method^49^.

### Native Gel Mobility Shift Assay

Native 10% Tris-Borate-EDTA (TBE) gels were prepared according to the published procedure^50^. Phospholamban was dissolved in binding buffer (0.1% C_12_E_8_, 20 mM Tris-HCl, 1 mM MgCl_2_, pH 7.0) and incubated with fluorescein-labeled ssDNA for 20 min at 25 °C. Samples were mixed at a 1:1 (v:v) ratio with ×2 loading buffer (Bromophenol blue (20% w/v), glycerol (50% v/v), 25 mM Tris-HCl, pH 7.0). Samples were run at 100 V at 4 °C and imaged by exposure to UV light. Signals were integrated using ImageJ densitometry software (National Institute of Mental Health, Bethesda, Maryland, USA). The *K*_d_ was determined from the non-linear fitting of the signal intensities versus PLN concentration^51^.

### FRET Experiments in reconstituted systems

SERCA was incubated with 1,5-I-AEDANS (Invitrogen), which specifically interacts with Cys 674 of the ATPase. The acceptor, Dabcyl-SE, was added at a 20-fold excess to WT PLN in 1% SDS, 100 mM NaHCO_3_, pH 9.0. The reaction was performed for 16 hours, and the labeled protein was purified using reverse phase HPLC. The steady-state fluorescence wavelengths were 350 nm (excitation) and 400–600 nm (emission).

### FRET in-cell Binding Assay

N-termini of canine SERCA2a and canine PLN were fused with either mCerulean (Cer), or enhanced yellow fluorescent protein (YFP)^23^. Phospholamban oligomerization, quaternary structure, and SERCA binding were measured by fluorescence resonance energy transfer in living cells (line AAV-293). Cells were cultured in complete DMEM growth medium with 10% fetal bovine serum, 1% L-glutamine and incubated at 37 °C under 5% CO_2_. The cultured cells were subjected to transient transfection using the MBS mammalian transfection kit (Stratagene, La Jolla, CA). Cells were co-transfected with plasmids encoding Cer-PLN-AFA and YFP-PLN-AFA, or Cer-SERCA and YFP-PLN-WT with a molar ratio of 1:5 or 1:20 respectively^26^, either in the presence or absence of 1 μM unlabeled 50-mer ssDNA. Following transfection, the cells were mildly trypsinized, resuspended in DMEM growth medium, plated on poly-D-lysine-coated glass bottom dishes, and allowed to adhere for 2 hours before imaging, as described previously^24^. The effect of ssDNA on PLN oligomerization and interaction with SERCA was quantified in live cells using wide-field fluorescence microscopy by acceptor sensitization FRET (EFRET) as described previously^24^,^52^. MetaMorph software was used to acquire a montage of 48 images using 40X objective having a numerical aperture of 0.75 and a motorized stage (Prior, Rockland, MA). Focus was automatically maintained by an optical feedback system (Perfect Focus System, Nikon). The exposure time was 150 ms for each channel: Cer, YFP, and FRET (Cer excitation/YFP emission). FRET efficiency was calculated according to the equation (4):


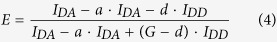


where: I_DD_ and I_AA_ are the fluorescence emission intensities of the donor channel (472/30 nm) with excitation of 427/10 nm, and the acceptor channel (542/27 nm) with excitation of 504/12 nm, respectively; I_DA_ is the fluorescence emission intensity of the FRET channel (542/27 nm) with excitation of 427/10 nm. The constants *a* and *d* are cross-talk coefficients determined from acceptor-only or donor-only control samples respectively. *G* represents the ratio of the sensitized emission to the corresponding amount of donor recovery. We obtained values of 0.085, 0.737 and 4.6 for *a*, *d*, and *G* ratio respectively.

The effect of ssDNA on parameters related to structure and binding affinity of PLN oligomer and SERCA-PLN regulatory complex were quantified by performing an ‘in-cell’ binding assay as described previously^24^. The FRET efficiency of individual cells co-expressing Cer-PLN-AFA and YFP-PLN-AFA, or Cer-SERCA and YFP-PLN-WT was plotted against relative protein concentration, quantified from the observed YFP fluorescence intensities. The cell-by-cell concentration dependence of FRET was fit to a hyperbolic curve to obtain the values of FRET_max_, K_D_1, and K_D_2. The maximal FRET (FRET_max_) is the intrinsic FRET of the protein complex, providing an estimate of average distances between the binding partners. K_D_ is the dissociation constant of the protein complex, providing an estimate of the apparent binding affinity. K_D_1 is the apparent dissociation constant of the PLN-PLN oligomer and K_D_2 is the apparent dissociation constant of the PLN-SERCA regulatory complex. For visualization, data from individual cells were pooled and the pooled data were fit by a hyperbolic or Hill function.

### Statistical Analysis

The data is pooled from 8 independent experiments for PLB-PLB FRET and 4 independent experiments for SERCA-PLB FRET using approximately 400–1200 cells per sample for each experiment. Errors are reported as standard error of the mean and statistical significance was evaluated using Student’s T test, where p < 0.05 was considered significant.

## Additional Information

**How to cite this article**: Soller, K. J. *et al.* Rheostatic Regulation of the SERCA/Phospholamban Membrane Protein Complex Using Non-Coding RNA and Single-Stranded DNA oligonucleotides. *Sci. Rep.* **5**, 13000; doi: 10.1038/srep13000 (2015).

## Supplementary Material

Supplementary Information

## Figures and Tables

**Table 1:** Random ssDNA sequences used.

**Sequence Length**	**Sequence 5′⟶3′**
5	FAM-GCT TG
10	FAM-ATA GCT TGC A
15	FAM-AGT GAT AGC TAT GGT
20	FAM-AGC AGC ACA GAG GTC AGA TG
30	FAM-ACT GAG CAT GGG ATA ACC GTT CTC AGA CTT
50	FAM-AGC AGC ACA GAG GTC AGA TGC AGG TAG GGT CCT ATG CGT GCT ACC GTG AA
80*	FAM-(N) _80_
80**	AGC AGC ACA GAG GTC AGA TGC AGG TAG GGT CCT ATG CGT GCT ACC GTG AAAGC AGC ACA GAG GTC AGA TGATA GCT TGC A

*A mixture of randomized 80-mer ssDNA sequences.

**80mer used for NMR.
