# Supplementary material for: Rheostatic Regulation of the SERCA/Phospholamban Membrane Protein Complex Using Non-Coding RNA and Single-Stranded DNA oligonucleotides
Source: Sci Rep. 2015 Aug 21;5:13000. doi: 10.1038/srep13000 (PMC4543939; doi:10.1038/srep13000)
Supplement: Supplementary Information [file srep13000-s1.doc]

**SUPPLEMENTARY INFORMATION**

**Rheostatic Regulation of the SERCA/Phospholamban Membrane Protein Complex Using Non-Coding RNA and Single-Stranded DNA oligonucleotides**

*Kailey J. Soller^1^, Raffaello Verardi^2^, Meng Jing^1^, Neha Abrol^3^, Jing Yang^1^, Naomi Walsh^2^, Vitaly V. Vostrikov^2^, Seth L. Robia^3^, Michael T. Bowser^1*^, and Gianluigi Veglia^1,2*^*

^1^Department of Chemistry, University of Minnesota, Minneapolis, Minnesota 55455.

^2^Department of Biochemistry, Molecular Biology, and Biophysics, University of Minnesota, Minneapolis, Minnesota 55455.

^3^Department of Cell and Molecular Physiology, Stritch School of Medicine, Loyola University Chicago, 60153

*Corresponding Author

Gianluigi Veglia

Department of Chemistry and Department of Biochemistry,

Molecular Biology and Biophysics

321 Church St. SE, Minneapolis, MN 55455

Telephone: (612) 625-0758

Fax: (612) 625-5780

E-mail: [vegli001@umn.edu](mailto:vegli001@umn.edu)

Michael T. Bowser
Department of Chemistry
312 Church St. SE, Minneapolis, MN 55455
Telephone: (612) 624-0873
Email: [bowser@umn.edu](mailto:bowser@umn.edu)


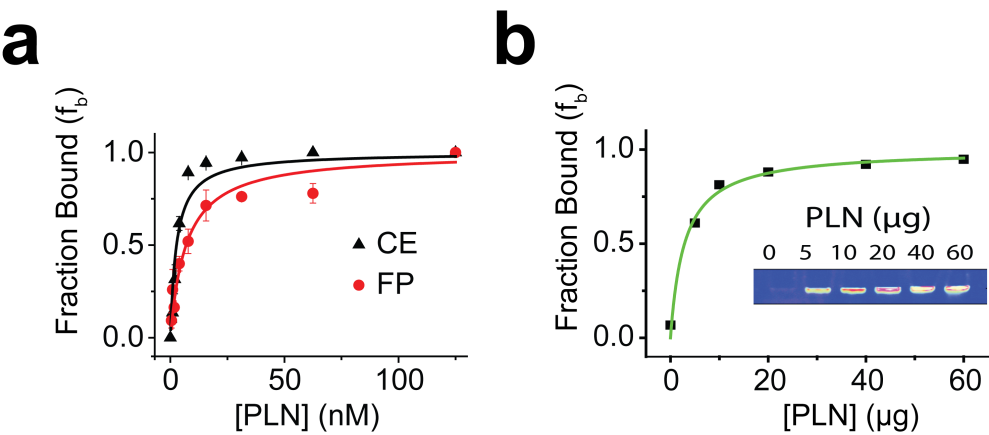


**Figure S1.** **ssDNA affinity for PLN as measured by FP, CE and native gel mobility shift**. (a) Binding curve of the 80mer (FAM labeled) ssDNA binding PLN as monitored by fluorescence polarization (*K*_d_ = 3.1 ± 0.8 nM) and affinity capillary electrophoresis (*K*_d_ = 0.52 ± 0.08 nM) (b) Binding curve of the 80mer (FAM labeled) ssDNA binding PLN as obtained from native gel mobility shift assays (*K*_d_ 2.9 ± 0.6 nM).

**
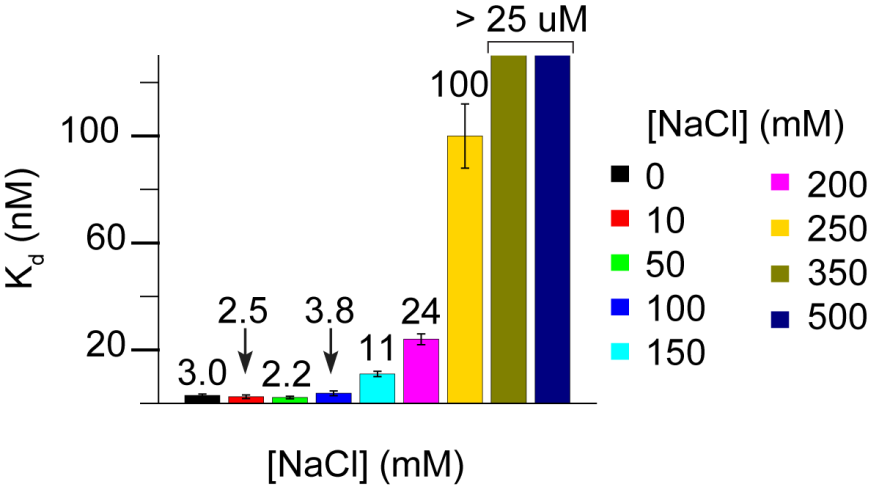
**

**Figure S2. Salt effect on ssDNA binding affinity for PLN.** Dissociation constants for ssDNA binding to PLN as measured by fluorescence polarization at different salt concentrations.

**
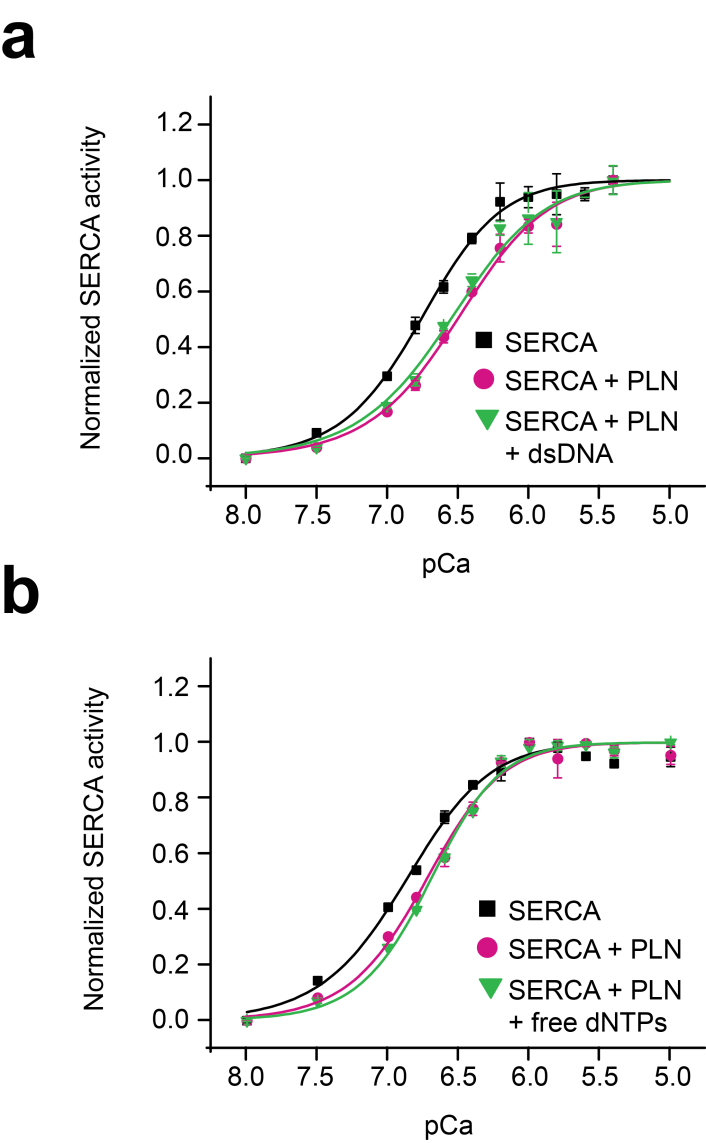
**

**Figure S3**. dsDNA (a) and free dNTPs (b) show no effect on SERCA activity.

**
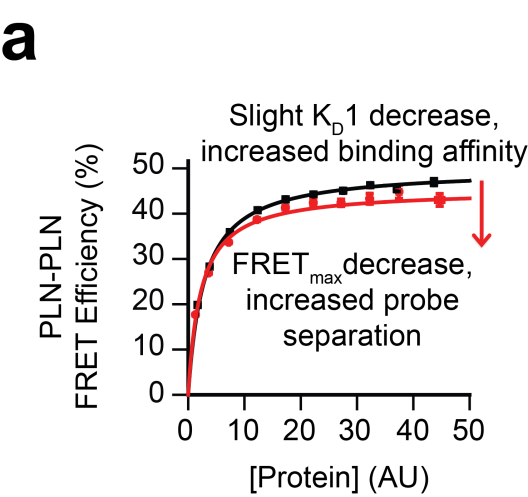
**

**Figure S4: FRET between PLN-PLN in HEK cells.** PLN-PLN FRET increased with protein expression up to a maximum (FRETmax). FRETmax decreased with addition of ssDNA, suggesting a transition away from a compact pentamer structure without loss of oligomerization.

**
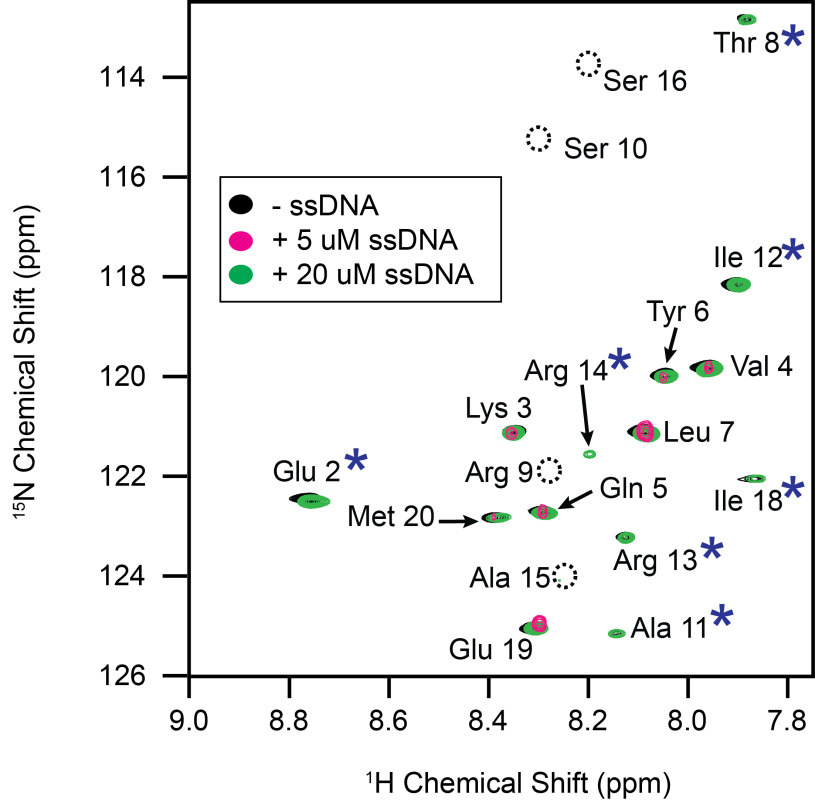
**

**Figure S5:** [^1^H, ^15^N] TROSY HSQC of PLN^AFA^ in the absence and in the presence of ssDNA**.**

**
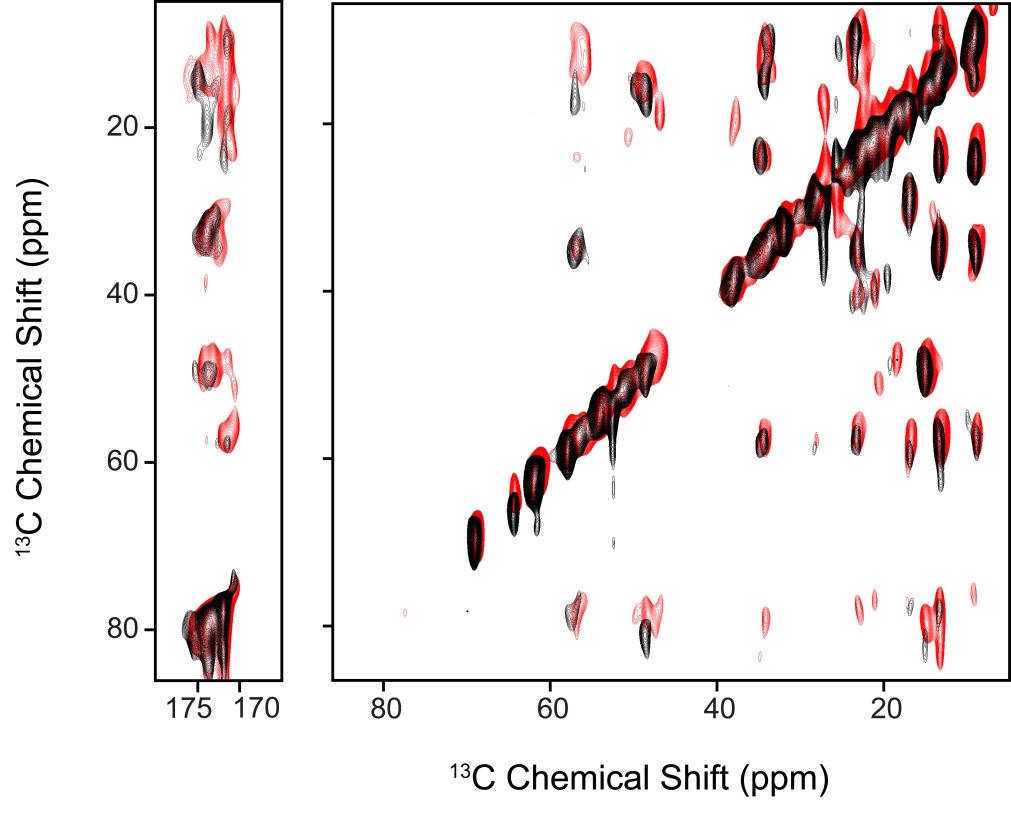
**

**Figure S6: [^13^C, ^13^C] Selectively labeled DARR of PLN^AFA^ with (red) and without (black) ssDNA.** 6 isotopically labeled sites are used here in the cytoplasmic domain of PLN^AFA^ including: Val4, Leu7, Ala11, Ala15, Ile 12, and Ile18. These spectra were collected at 20°C with a 200 msec mixing time.
